# Local Temporal Correlation Common Spatial Patterns for Single Trial EEG Classification during Motor Imagery

**DOI:** 10.1155/2013/591216

**Published:** 2013-11-20

**Authors:** Rui Zhang, Peng Xu, Tiejun Liu, Yangsong Zhang, Lanjin Guo, Peiyang Li, Dezhong Yao

**Affiliations:** Key Laboratory for Neuroinformation of Ministry of Education, School of Life Science and Technology, University of Electronic Science and Technology of China, Chengdu 610054, China

## Abstract

Common spatial pattern (CSP) is one of the most popular and effective feature extraction methods for motor imagery-based brain-computer interface (BCI), but the inherent drawback of CSP is that the estimation of the covariance matrices is sensitive to noise. In this work, local temporal correlation (LTC) information was introduced to further improve the covariance matrices estimation (LTCCSP). Compared to the Euclidean distance used in a previous CSP variant named local temporal CSP (LTCSP), the correlation may be a more reasonable metric to measure the similarity of activated spatial patterns existing in motor imagery period. Numerical comparisons among CSP, LTCSP, and LTCCSP were quantitatively conducted on the simulated datasets by adding outliers to Dataset IVa of BCI Competition III and Dataset IIa of BCI Competition IV, respectively. Results showed that LTCCSP achieves the highest average classification accuracies in all the outliers occurrence frequencies. The application of the three methods to the EEG dataset recorded in our laboratory also demonstrated that LTCCSP achieves the highest average accuracy. The above results consistently indicate that LTCCSP would be a promising method for practical motor imagery BCI application.

## 1. Introduction

Brain-computer interfaces (BCI) use electroencephalographic signals or other electrophysiological measures of brain activity to provide a new nonmuscular channel for sending messages and commands to the external world. According to the different electrophysiological signals which they use, BCI can fall into 6 groups [1]: visual evoked potentials (VEP) based BCI [2]; slow cortical potentials (SCP) based BCI [3]; evoked potentials P300 based BCI [4]; mu and beta rhythms (ERD/ERS) based BCI [5], cortical neuronal action potentials based BCI [6] and hybrid BCI [7]. Among them, ERD/ERS based BCI has received a lot of attentions in recent years due to its potential application in motor rehabilitation and its assisting for the motor function impaired patients [8–10]. 

Feature extraction and classification algorithms play important roles for the performance of ERD/ERS based BCI, and there are various methods have been proposed to extract ERD/ERS related features [11, 12], such as the laplacian method [13], autoregressive spectral analysis [14], common spatial pattern (CSP) [15], discriminative spatial patterns [16], bispectrum analysis [17], and multivariate empirical mode decomposition [18]. Currently, CSP is one of the most popular feature extraction methods for ERD/ERS based BCI, its efficiencies have been proved by the BCI competitions [19, 20] and various ERD/ERS based BCI studies [21–23]. Besides BCI discipline, CSP has been also applied to normal versus abnormal EEGs classification [24] and EEG source localizations [25]. 

The aim of CSP is to learn the optimal spatial filters which maximize the variance of one class while minimizing the variance of the other class simultaneously [26, 27]. Mathematically, CSP relies on the simultaneous diagonalization of two covariance matrices. However, there are two inherent drawbacks for the estimation of the covariance matrices in using the conventional strategy; on one hand, it is prone to be influenced by outlier noise; even one outlier may make the spatial filters obtained meaningless [28]; on the other hand, the temporal information is neglected, while the time-dependent local variances may deliver more discriminant power than the global variances [29]. To deal with above shortcomings in conventional CSP, Local Temporal Common Spatial Patterns (LTCSP) propose to use the Euclidean distance between *x*_*l*_ and *x*_*m*_ (both *x*_*l*_ and *x*_*m*_ are *N*-channel EEG recording vectors at two given time points *l* and *m*) as a weight to emphasize the covariance matrices [29]. From the perspective of neurophysiology, the task related signal is usually hidden in strong spontaneous brain background activity, which is nonstationary  [30, 31]. Therefore, the scalp-recorded signal is very noisy even if the recordings are from high-performance EEG amplifier with suitable reference strategy [32, 33] and in a finely shielded room with a very cooperative subject. In this case, the Euclidean distance measure may give unsuitable weight coefficient due to the noise effect. Besides, the Euclidean distance is not convergent with distribution in a wide range, which may reduce the robustness of LTCSP. In this work, the correlation measure was newly proposed to replace the Euclidean distance in order to solve the above problems. In fact, when Müller-Gerking et al. firstly introduced CSP into BCI areas [26], the correlations of neighboring electrodes had been argued as an important information which implies the need to pay attention for them.

The framework of this paper is arranged as follows.  Section 2 gives details about LTCSSP; In Section 3, CSP, LTCSP, and LTCCSP are evaluated on simulated outliers influenced BCI Competition datasets. Then the comparison results of the above three methods on the actual EEG dataset recorded in our lab are given in Section 4; Sections 5 and 6 include discussion and final conclusion, respectively. Besides the above sections, an appendix was provided to describe the mathematical details of CSP.

## 2. Materials and Methods

### 2.1. Principles of LTCCSP

LTCCSP is an extension of the conventional CSP (See Appendix for detail). The spatial filter matrix Γ of conventional CSP is obtained by maximizing the variance of one class while minimizing the variance of the other class simultaneously. We denote *N* × *S* matrix *X* and *Y* as the EEG data under task is 1 and 2, with  *N*  being the number of channels and *S* being the number of samples in each trial. Formally, the object function of CSP could be expressed as [34]

(1) max⁡ ΓTR−XΓΓTR−YΓs.t. ΓTR−YΓ=I,

where ${\overset{-}{R}}_{X}$ and ${\overset{-}{R}}_{Y}$ are the average normalized spatial covariance matrices of tasks 1 and 2 and Γ is the spatial filter matrix. Generally, the first and last few columns of Γ are served as optimized spatial filters. 

Then taking the first *M* columns of Γ for an example, (1) could be transformed to

(2) ΓTR−XΓΓTR−YΓ=ΓT(1/KX∑i=1KXRX(i))ΓΓT(1/KY∑i=1KYRY(i))Γ=1/KX∑i=1KX∑j=1M(γjTX(i)X(i)Tγj)/tr⁡(X(i)X(i)T)1/KY∑i=1KY∑j=1M(γjTY(i)Y(i)Tγj)/tr⁡(Y(i)Y(i)T),

where *K*_*X*_ and *K*_*Y*_ are, respectively, the numbers of trials under each task, (*i*) denotes the *i*th trial, and *γ*_*j*_ is the *j*th column of the matrix Γ. The last *M* columns could be transformed as above, too. 

Using the dimension reduction strategy in [35], the quadratic forms *γ*_*j*_^*T*^ *XX* ^*T*^ *γ*_*j*_  could be transformed to

(3) $$\begin{array}{c} \gamma_{j}^{T}XX^{T}\gamma_{j}=\frac{1}{2S}\overset{S}{\underset{l=1}{\sum }}\overset{S}{\underset{\;m=1}{\sum }}{\left(\gamma_{j}^{T}x_{l}-\gamma_{j}^{T}x_{m}\right)}^{2}, \end{array}$$

where *x*_*l*_ and *x*_*m*_ are the data vectors at time points *l* and *m*, respectively. Obviously, (3) mainly focuses on the global information of trials. However, in actual BCI situation, the local information may be helpful for task recognition. Therefore, the weight matrix can be added to emphasize the local information as follows [29]:

(4) $$\begin{array}{c} \frac{1}{2S}\overset{S}{\underset{l=1}{\sum }}\overset{S}{\underset{m=1}{\sum }}{\left(\gamma_{j}^{T}x_{l}-\gamma_{j}^{T}x_{m}\right)}^{2}W_{lm}^{X}, \end{array}$$

where *W*_*lm*_^*X*^ is the weight matrix. The fundamental derivation of weight matrix is that if the two concerned patterns *x*_*l*_ and *x*_*m*_ are close, it will impose a large coefficient in the weight matrix. Hence, we will use the correlation coefficient to define the corresponding weight matrix as follows:

(5) $$\begin{array}{c} W_{lm}^{X}=\{\begin{array}{cc} \exp\left(\text{corr}\left(x_{l},x_{m}\right)\right), & \left|l-m\right|<\tau \\ 0, & \text{otherwise}, \end{array} \end{array}$$

where corr( ) denotes the correlation coefficient operator and *τ* is the local temporal range. Obviously, the weight matrix will impose a relatively larger coefficient on the similar patterns in the concerned local temporal range *τ*. 

 Moreover, (4) will be further expressed as

(6)   12S∑l=1S∑m=1S(γjTxl−γjTxm)2WlmX  =γjT(12S∑l=1 S∑m=1S(xl−xm)(xl−xm)TWlmX)γj  =γjT(1S(∑l=1 S∑m=1SxlxlTWlmX−∑l=1S∑m=1SxlxmTWlmX))γj  =γjT(1S(XDXXT−XWXXT))γj  =γjT(1SXLXXT)γj,

where the Laplacian matrix *L* ^*X*^ = *D* ^*X*^ − *W* ^*X*^ and *D* ^*X*^ is a diagonal matrix with the diagonal elements being the row sums of *W* ^*X*^; that is, *D*_*ll*_^*X*^ = ∑_*m*=1_^*S*^ *W*_*lm*_^*X*^. Equation (6) is also held for EEG trials *Y* under task 2. Using (6), (2) can be converted as follows:

(7) (1KX∑i=1KX∑j=1M(γjTX(i)LX(i)X(i)Tγj)tr⁡(X(i)LX(i)X(i)T))  ×(1KY∑i=1KY∑j=1M(γjTY(i)LY(i)Y(i)Tγj)tr⁡(Y(i)LY(i)Y(i)T))−1,

where tr⁡(*X*(*i*)*L* ^*X*^(*i*)*X*(*i*)^*T*^) and tr⁡(*Y*(*i*)*L* ^*Y*^(*i*)*Y*(*i*)^*T*^) are used to replace tr⁡(*X*(*i*)*X*(*i*)^*T*^)   and tr⁡(*Y*(*i*)*L* ^*Y*^(*i*)*Y*(*i*)^*T*^), respectively. Let ${\tilde{R}}_{X}=(1/K_{X})\sum_{i=1}^{K_{X}}\left(\left(X(i)L^{X}(i)X{\left(i\right)}^{T}\right)/\left(\mathrm{tr}(X(i)L^{X}(i)X{\left(i\right)}^{T})\right)\right)$ and ${\tilde{R}}_{Y}=\left(1/K_{Y}\right)\sum_{i=1}^{K_{Y}}\left(\left(Y(i)L^{Y}(i)Y{\left(i\right)}^{T}\right)/\left(\mathrm{tr}(Y(i)L^{Y}(i)Y{\left(i\right)}^{T})\right)\right)$ be the average normalized local temporal correlation covariance matrices of two classes; (7) can be simplified to

(8) $$\begin{array}{c} \frac{\Gamma^{T}{\tilde{R}}_{X}\Gamma }{\Gamma^{T}{\tilde{R}}_{Y}\Gamma }. \end{array}$$

Noting that (8) has the same structure as (1), so the solution of maximizing (8) subject to $\Gamma^{T}{\tilde{R}}_{Y}\Gamma =I$ is similar to (1), and the spatial filter matrix could be given by

(9) $$\begin{array}{c} \tilde{\Gamma }=\tilde{U}{\tilde{D}}^{-1/2}\tilde{V}, \end{array}$$

where $\tilde{U}$ is the matrix of eigenvectors of ${\tilde{R}}_{X}+{\tilde{R}}_{Y}$ with $\tilde{D}$ being the diagonal matrix of associated eigenvalues and $\tilde{V}$ is the eigenvectors matrix of ${\tilde{D}}^{-1/2}{\tilde{U}}^{T}{\tilde{R}}_{X}\tilde{U}{\tilde{D}}^{-1/2}$.

### 2.2. Online Implementation of LTCCSP

In the online application, when the spatial filter matrix $\tilde{\Gamma }$ is obtained from the training dataset, there also exists a little difference between LTCCSP and conventional CSP for feature extraction. Take *L* as a generic symbol for *L* ^*X*^(*i*) and *L* ^*Y*^(*i*); since *L* is a semipositive definite matrix, it can be decomposed as *L* = *L* ^1/2^(*L* ^1/2^)^*T*^. Then EEG data *C*  from a trial  should be spatially filtered as $\tilde{Z}=\tilde{\Gamma }CL^{1/2}$. At last, the logarithm variances of the first and last *M*  rows of $\tilde{Z}$ are served as final features and will be sent to classifier for classification.

## 3. Evaluations on Simulated Outliers Influenced EEG Datasets

### 3.1. EEG Datasets Description

*(1) Dataset IVa of BCI Competition III*. This dataset contains EEG signals recorded from five subjects by using 118 electrodes [36]. In each trial, a visual cue was shown for 3.5 s, during which three kinds of motor imageries were performed, that is, left hand, right hand, and right foot. The motor imageries of right hand and foot were needed to be classified. The total number of EEG trials for each subject is 280. In particular, 168, 224, 84, 56, and 28 trials are used as training data corresponding to the five subjects: aa, al, av, aw, and ay, respectively. The data were band-pass filtered between 0.05 and 200 Hz and downsampled at 100 Hz for subsequent analysis.

*(2) Dataset IIa of BCI Competition IV.* The EEG data was recorded from nine subjects, who were asked to perform four different kinds of motor imagery tasks, that is, left hand, right hand, foot, and tongue [37]. Two sessions recorded on different days were made available for each individual, and each session consisted of 288 trials with 72 trials per class. The EEG signals, measured by 22 electrodes, were sampled at 250 Hz. In our experiment, one session containing the class labels for all trials is used as training set and the unlabelled session as test set. We focus on two-class classification scenario, that is, classifying EEG signals belonging to left and right hand motor imageries.

### 3.2. Preprocessing

Following the winner of BCI competition IV and [34], the EEG segments recorded from 0.5 s to 3.75 s after the visual cue were used for subsequent analysis on the first dataset, and the time interval from 0.5 s to 2.5 s was used on the second dataset. Then the EEG segments were band-pass filtered between 8 Hz and 30 Hz.

### 3.3. Introducing Outliers

We introduced outliers into the two datasets from BCI Competitions with the aim to simulate the actual recordings with outliers contamination and then quantitatively compare the classification accuracies of the conventional CSP and LTCSP methods and the proposed LTCCSP method. For each subject, the outliers were generated by the one-dimensional Gaussian distribution *N*(*μ* + 30*σ*; 30^2^ *σ* ^2^), where *μ* and *σ* are the mean and standard deviation of the training EEG segments, respectively. The generated outliers were added to the training EEG data to simulate polluted EEG signals. The number of outliers is varying from 0 to 0.4*n* with step 0.1*n*, where *n* is the number of trials in training set. We randomly selected the time positions to add the outliers.

### 3.4. Results

We used CSP, LTCSP, and LTCCSP to extract the motor imagery-related features, respectively. Comparing with CSP, there are two parameters in LTCSP and one parameter in LTCCSP that need to be configured. We set *σ* = 7*σ*_0_ in using LTCSP, where *σ*_0_ is the standard deviation of the squared norms of the training samples, as the highest average classification accuracy was achieved under this parameter [29]. For the local temporal range *τ* that exists in both LTCSP and LTCCSP, we used 10-fold cross-validation method to compare the average accuracies of *τ* within {2,…, 12} on the training set and then set *τ* as the one corresponding to the highest average accuracy. 

Three pairs of spatial filters were used for feature extraction for all of the three CSP-based methods, as recommended in [15]. Then the log-variances of the spatially filtered EEG signals were used as input features for a classifier. Support Vector Machine (SVM), one of the most popular classifiers in BCI application, was used for classification [38], and the default parameters were set for SVM. The experiments were repeated ten times for each occurrence frequency of outliers; the average accuracies and the mean accuracies for all of the subjects were reported in Table 1.

All three CSP-based methods achieved the highest classification accuracies on the clean EEG data compared to those cases with various occurrence frequencies of outliers added. When outliers were introduced, the performance of all the three methods was deteriorated with the increasing occurrence frequencies of outliers for most of the subjects except some subjects, that is, *Sub ay, Sub 1, *and* Sub 2*. For *Sub 2*, all the three methods achieved approximately 50% accuracies, which may indicate BCI illiteracy phenomenon [39], that is, the subject cannot control BCI effectively by motor imagery. 

Table 1 also revealed that both LTCCSP and LTCSP achieve better performance than the conventional CSP under various outliers conditions, where LTCCSP showed the statistically significant improvement but not for LTCSP. Among the three methods, the best performance was achieved by LTCCSP on both clean EEG datasets and outliers influenced EEG datasets.

## 4. Evaluation on Actual EEG Dataset

### 4.1. EEG Datasets Description

*Dataset Recorded in Our Laboratory. *This dataset consists of EEG data from 13 subjects (11 males and 2 females, right handed, 19–25 years old). The experimental protocol was approved by the Institution Research Ethics Board at University of Electronic Science & Technology of China. All participants were asked to read and sign an informed consent form before participating in the study. After experiment, all the participants received a monetary compensation for their time and effort. Subjects sat in a comfortable armchair in front of a computer screen; they were asked to perform motor imagery with left hand or right hand according to the instructions appeared on the screen. Motor imagery lasted for 5 seconds, and followed a 5 seconds rest. Fifteen Ag/AgCl electrodes covers sensorimotor area were used for EEG recordings with Symtop Amplifier (Symtop Instrument, Beijing, China), and the signals were sampled with 1000 Hz and band-pass filtered between 0.5 Hz and 45 Hz. Four runs were collected; each run consisted of 50 trials, 25 trials for each class, and there was a 3-minute break between the consecutive two runs. The first two runs are served as training dataset, and the last two runs are served as test dataset.

### 4.2. Preprocessing

All the EEG segments during motor imagery period were selected, that is, from 0 s to 5 s. Then the selected EEG segments were band-pass filtered between 8 Hz and 30 Hz.

### 4.3. Results

We used CSP, LTCSP, and LTCCSP to extract the motor imagery related features, respectively, and the parameters of LTCCSP and LTCSP were the same as we mentioned in Section 3.4. SVM with the default parameters was used for classification. The classification accuracy for each subject and the mean accuracy were reported in Table 2. 

On the EEG dataset recorded in our lab, LTCCSP achieved the highest mean accuracy across the three methods. As revealed in Table 2, the highest accuracies were achieved by LTCCSP in 11 of 13 subjects. Furthermore, when we set the accuracies obtained by CSP as the baseline; the accuracies obtained by LTCCSP are higher or at least equal to the baseline, while the accuracies obtained by LTCSP are not consistently improved across the 13 subjects. Paired *t*-test showed that there is statistical difference between CSP and LTCCSP, while no statistical difference existed between CSP and LTCSP. 

## 5. Discussions

Both LTCCSP and LTCSP introduce local temporal information to the covariance matrices estimation procedures in CSP, and our experiments indicate that the local temporal information is effective in handling outliers. If we take the performance of CSP as baseline, Table 1 indicates that LTCCSP consistently improves the performance for most of subjects under outliers condition, but the improvement of LTCSP under the same situation is not stable. Table 1 also tells us that the performance of LTCSP is worse than that of CSP on the two clean BCI Competition datasets without outliers introduced, but Table 2 shows us that LTCSP achieves better performance than CSP on the EEG dataset recorded in our lab.

We think that the performance difference between LTCCSP and LTCSP is mainly caused by the definition of the weight matrix. LTCCSP uses correlation measure to construct the weight matrix, while LTCSP uses the Euclidean distance to construct the weight matrix. In our opinion, the Euclidean distance measure is not good enough because sometimes it will wrongly treat normal signal as noise signal. An extreme case is that, for example, *x*_*l*_ and *x*_*m*_, where *x*_*m*_ = *x*_*l*_ + constant vector, are EEG data vectors selected from time points *l* and *m*, respectively. When we calculate the Euclidean distance of them, we will get the Euclidean norm of the constant vector. Obviously, if the constant vector is of a large norm, the Euclidean distance of them will be large too, then, in the framework of LTCSP the corresponding EEG information will be strongly suppressed by imposing a very small weight on the estimation of the covariance matrix. However, no matter how big is the norm of the constant vector, the correlation coefficient of them is fixed at 1. In practical application, similar situations may appear in natural EEG signals due to the transient baseline shift. In addition, since the crucial concern of CSP is based on the spatial pattern [26], the data vectors that have similar spatial patterns should be imposed with a relatively large weight. Therefore, correlation coefficient may be a more suitable measure to construct the weight matrix.

In summary, compared with the Euclidean distance-based LTCSP, correlation-based LTCCSP may provide the following merits.

1. The correlation coefficient distributes in [−1,1], while the Euclidean distance distributes in a wide range, which makes LTCCSP more stable than LTCSP.
2. Use of correlation may reduce the possibility of imposing small weight to natural EEG signal.
3. There is only one parameter *τ* in LTCCSP, while in LTCSP there are two parameters need to configure, *τ* and *σ*, which makes LTCCSP easier to implement in practical use.

It should be noted that several methods have also been proposed to improve CSP's performance. For example, adaptive CSP is developed to deal with time-varying signals [40]; nonlinear CSP overcomes the linearity restriction [41]; L1 norm CSP enhances its robustness [42]. Besides, an excellent method named extreme energy ratio (EER) has been recently proposed [43, 44], which also relies on covariance matrices estimation and eigenvalue decomposition, but it aims at maximizing or minimizing the disparity of energy features between two classes of EEG signals. Afterwards, semisupervised EERs are developed to solve the small training set and time-varying problems in BCI application [45]. The algorithm proposed in this paper is mainly to establish robust covariance matrices estimation by introducing local temporal correlation information, and it could be complementary to the above existing methods to develop the more competitive method in the future.

## 6. Conclusions

In this paper, we proposed a practical feature extraction method named LTCCSP, which considers local temporal correlation information in the learning process of the conventional CSP, for optimizing spatial filters. The current results confirmed that LTCCSP has the ability to obtain meaningful spatial filters from natural EEG data and noise influenced EEG data. Furthermore, LTCCSP is simple for application as there is only one parameter that needs to be configured. However, it should be noted that though LTCCSP shows its effective ability for motor imagery-related feature extraction, it still cannot completely suppress the introduced noise, and much work is still needed to promote for a more robust feature extraction.

## Figures and Tables

**Table 1:** Average classification accuracies (%) of the Dataset IVa of BCI Competition III and Dataset IIa of BCI Competition IV for CSP, LTCSP, and LTCCSP with increasing occurrence frequencies of outliers.

Freq		Dataset IVa of BCI Competition III					Dataset IIa of BCI Competition IV									
	Subject	aa	al	av	aw	ay	1	2	3	4	5	6	7	8	9	Mean
0	CSP	75.0	100.0	69.9	91.5	74.2	84.0	54.5	95.8	75.3	58.0	68.1	80.6	92.4	93.7	79.5
	LTCSP	63.4	96.4	61.7	75.9	49.6	88.8	58.5	95.1	73.9	70.0	72.3	81.3	95.8	92.3	76.8
	LTCCSP	77.7	100.0	73.0	92.9	78.2	90.9	60.2	96.5	78.2	72.9	70.2	81.3	95.1	93.7	82.9*

0.1	CSP	70.5	100.0	68.2	86.1	76.5	86.9	56.2	93.5	65.9	56.1	61.7	66.0	92.2	92.7	76.6
	LTCSP	65.2	98.8	61.8	77.5	49.1	87.8	56.8	94.5	69.4*	69.5*	71.6*	80.4*	97.5*	92.4	76.6
	LTCCSP	74.0*	100.0	71.9*	91.6*	78.5*	91.7*	61.1*	95.5*	72.0*	64.7*	66.4*	71.9*	95.2*	93.7*	80.6*

0.2	CSP	69.6	98.9	68.0	89.2	76.3	86.3	56.0	93.4	64.8	51.7	61.8	62.6	93.1	92.5	76.0
	LTCSP	64.6	97.7	62.1	75.2	49.4	88.3*	59.1*	94.8*	68.9*	69.1*	71.3*	80.2*	97.3*	92.5	76.5
	LTCCSP	73.6*	99.8*	70.2*	92.8*	79.9*	90.6*	61.9*	95.7*	69.5*	59.1*	67.5*	69.4*	94.7*	93.6*	79.9*

0.3	CSP	67.6	99.8	69.3	86.6	77.7	87.1	55.9	93.0	64.0	51.9	62.2	60.1	94.2	92.2	75.8
	LTCSP	63.2	98.9	63.0	74.2	49.2	87.6	58.6	94.1*	67.5*	69.1*	70.1*	81.3*	97.4*	92.5	76.2
	LTCCSP	71.2*	100.0	73.3*	91.9*	81.4*	89.8*	60.0*	94.9*	68.0*	58.9*	67.2*	65.5*	95.6*	93.6*	79.4*

0.4	CSP	58.7	99.6	61.7	82.4	74.0	85.3	53.7	93.1	65.1	53.9	61.0	58.6	93.3	92.5	73.8
	LTCSP	64.6*	97.7	63.1*	72.0*	49.0	87.9*	58.1*	94.3*	67.8*	67.4*	69.5*	81.5*	97.0*	92.5	75.9
	LTCCSP	67.3*	99.8	67.8*	86.6*	77.7*	89.2*	59.3*	95.1*	67.7*	56.8*	65.8*	64.3*	95.0*	93.8*	77.6*

*Paired *t*-test *P* < 0.05 between two concerned methods, that is, LTCSP versus CSP and LTCCSP versus CSP. The bold values indicate the best performance among the three methods.

**Table 2:** Classification accuracies (%) of the Dataset recorded in our laboratory for CSP, LTCSP, and LTCCSP.

Subject	TCY	LPY	GK	WCF	WZQ	CR	HYR	ZB	PKH	FNX	XJP	WXY	DT	Mean
CSP	57.0	79.0	94.0	81.0	69.0	76.0	77.0	68.0	79.0	72.0	97.0	72.0	82.0	77.2
LTCSP	65.0	73.0	98.0	79.0	69.0	83.0	79.0	72.0	82.0	63.0	98.0	77.0	80.0	78.3
LTCCSP	65.0	79.0	95.0	83.0	78.0	83.0	79.0	70.0	87.0	75.0	98.0	77.0	82.0	80.8*

*Paired *t*-test *P* < 0.05 between two concerned methods, that is, LTCSP versus CSP and LTCCSP versus CSP. The bold values indicate the best performance among the three methods.

## Appendix

### The Method of Common Spatial Patterns

We describe here the mathematical part of the method of CSP as used in the present paper [26].

Let *N* × *S* matrix *X* denote the filtered data of a trial under task 1, with *N* being the number of channels and *S* being the number of samples in time. Thus, the recording at a given time point can be represented as a point in *N*-dimensional Euclidean space and also can be seen as a spatial pattern. The normalized spatial covariance matrix is calculated as

(A.1) $$\begin{array}{c} R_{X}=\frac{XX^{T}}{\text{trace}\left(XX^{T}\right)}. \end{array}$$

Likewise, let *R*_*Y*_ denote the corresponding normalized spatial covariance matrix of a trial under task 2. Then, the normalized spatial covariance matrices are averaged over trials, ${\overset{-}{R}}_{X}$ and ${\overset{-}{R}}_{Y}$ are obtained for each task. Next, whiten the composite spatial covariance matrix ${\overset{-}{R}}_{X}+{\overset{-}{R}}_{Y}$; that is, determine a matrix *P* such that

(A.2) $$\begin{array}{c} P^{T}\left({\overset{-}{R}}_{X}+{\overset{-}{R}}_{Y}\right)P=I, \end{array}$$

where *I* is an identity matrix. The whitening matrix is formed as *P* = *UD* ^−1/2^, where *U* is the eigenvectors matrix of ${\overset{-}{R}}_{X}+{\overset{-}{R}}_{Y}$ and *D* is the diagonal matrix of associated eigenvalues. After that, let $G_{X}=P^{T}{\overset{-}{R}}_{X}P$ and $G_{Y}=P^{T}{\overset{-}{R}}_{Y}P$, respectively; then *G*_*X*_ and *G*_*Y*_ share the same eigenvectors matrix *V*:

(A.3) $$\begin{array}{c} V^{T}G_{X}V=\Lambda ,\;\;V^{T}G_{Y}V=I-\Lambda .\; \end{array}$$

This decomposition can be accomplished due to *G*_*X*_ + *G*_*Y*_ = *I*. Make the eigenvalues mentioned are sorted in descending order; in consequence, the final optimal spatial filter is given by

(A.4) $$\begin{array}{c} \Gamma =PV. \end{array}$$

Using this projection matrix Γ, the EEG signals *X* and *Y* are projected as

(A.5) $$\begin{array}{c} Z_{X}=\Gamma^{T}X,\;\;Z_{Y}=\Gamma^{T}Y. \end{array}$$

Since the sum of the corresponding eigenvalues is always one, the variances of first and last few rows of *Z*_*X*_ and *Z*_*Y*_ are suitable features for classification.
